# *Porphyromonas gingivalis* infection induces lysine lactylation reprogramming in human umbilical vein endothelial cells

**DOI:** 10.3389/fcimb.2026.1706727

**Published:** 2026-01-30

**Authors:** Qinrui Wu, Zhengyi Li, Tao Gong, Xin Zheng, Xuedong Zhou, Xian Peng

**Affiliations:** 1 State Key Laboratory of Oral Diseases and National Center for Stomatology and National Clinical Research Center for Oral Diseases, West China Hospital of Stomatology, Sichuan University, Chengdu, China; 2 Department of Dental and Endodontic Diseases, West China Hospital of Stomatology, Sichuan University, Chengdu, China

**Keywords:** cardiovascular diseases, endothelial dysfunction, lysine lactylation, periodontal diseases, *Porphyromonas gingivalis*

## Abstract

**Introduction:**

*Porphyromonas gingivalis* (Pg), a keystone periodontal pathogen, is a known risk factor for atherosclerosis and cardiovascular disease. Lysine lactylation (Kla) is an emerging post-translational modification (PTM) that bridges cellular metabolism and epigenetic regulation. However, the involvement of Kla in bacteria-induced endothelial dysfunction remains unexplored. This study aims to characterize the global lactylation landscape in human umbilical vein endothelial cells (HUVECs) following Pg infection.

**Methods:**

HUVECs were infected with Pg, and their lactylome was analyzed using LC-MS/MS-based quantitative proteomics. Differentially lactylated sites were identified based on a fold change (FC) of ≥ 1.5 or ≤ 0.67 with a significance level of p < 0.05. Bioinformatics tools, including pathway enrichment and protein-protein interaction (PPI) network analyses, were employed to determine the biological significance of the modified proteins.

**Results:**

A total of 5,788 Kla sites were identified across 1,881 proteins. Following Pg infection, 487 sites were significantly upregulated and 598 sites were downregulated. Functional enrichment analysis revealed that differentially lactylated proteins are primarily involved in nucleocytoplasmic transport, bacterial invasion, ribosome biogenesis, and DNA repair mechanisms. Network analysis highlighted five highly interconnected clusters regulating translation, RNA processing, and metabolism. Notably, key endothelial structural and regulatory proteins, including AHNAK (160 sites), MYH9 (56 sites), and FLNA (34 sites), exhibited extensive lactylation.

**Discussion:**

This study provides the first comprehensive lactylome profile of Pg-infected HUVECs, identifying lysine lactylation as a novel mechanism linking periodontal infection to endothelial dysfunction. These findings offer a new molecular framework for understanding the pathogenesis of periodontitis-associated cardiovascular diseases and suggest potential biomarkers and therapeutic targets.

## 1 Introduction

Periodontitis, a chronic bacterial infection of the oral cavity, is a globally prevalent disease that affects millions of people each year and leads to the progressive degradation of periodontal tissues and eventual tooth loss (Orlandi et al., 2022; Heitz-Mayfield, 2024; Chen et al., 2021b). *Porphyromonas gingivalis* (Pg), a Gram-negative, obligately anaerobic rod-shaped bacterium, is one of the most prevalent microbial species in subgingival plaque and is strongly associated with periodontal pathology (Reyes, 2021; Shiheido-Watanabe et al., 2023). This pathogen, along with its virulence factors, can enter the bloodstream via breached gingival connective tissue-whether due to periodontal destruction or oral clinical treatments (Orlandi et al., 2022)-and is capable of circulating to reach distant organs (Schenkein et al., 2020). Consequently, Pg is not only a key etiological agent of periodontitis but has also been implicated in the pathogenesis of several systemic conditions, including cardiovascular disease (Miyauchi et al., 2025; Huang et al., 2025), Alzheimer’s disease (Dominy et al., 2019; Liu et al., 2024), and rheumatoid arthritis (Ahmadi et al., 2023).

From the early 2000s, considerable attention has been focused on the association between Pg and cardiovascular diseases (CVDs) (Li et al., 2002). CVDs encompass a spectrum of chronic conditions such as coronary heart disease, stroke, congestive heart failure, and peripheral artery disease (Kholy et al., 2015). These disorders represent major public health concerns due to their potential to precipitate life-threatening acute clinical events, including myocardial infarction and stroke (Kholy et al., 2015; Chong et al., 2025). While traditional risk factors such as hypercholesterolemia, hypertension, smoking, and high sodium intake remain fundamental to disease development, recent evidence highlights the increasingly prominent role of chronic inflammation in the initiation and progression of atherosclerosis (Schenkein et al., 2020). This growing body of research underscores the need to explore novel inflammatory triggers, including infectious agents such as Pg, in the pathogenesis of cardiovascular disorders.

Protein translational modifications (PTMs) refer to covalent alterations that occur after protein synthesis, either through proteolytic cleavage or the attachment of functional groups to specific amino acid residues (Gao et al., 2024). These modifications regulate key aspects of protein function, including activity, subcellular localization, stability, and molecular interactions, thereby playing critical roles in cellular signaling pathways (Biffo et al., 2024). One notable example is the recently identified lysine lactylation (Kla), a type of protein modification that originates from lactate. This modification has been demonstrated to occur on core histones in a p300-dependent mechanism (where p300 acts as the principal enzyme catalyzing Kla) (Zhang et al., 2019). Emerging evidence indicates that histone Kla serves as an epigenetic regulator that promotes the expression of M2-type genes-a set of genes associated with anti-inflammatory and tissue-repair functions in macrophages under bacterial challenge, thereby contributing to the maintenance of immune homeostasis (Yang et al., 2023; Zhang et al., 2019). Proper lysine Kla plays a crucial role in regulating essential physiological processes, such as cellular energy adaptation and stress-responsive gene expression (Zhang et al., 2019). However, growing evidence indicates that dysregulated Kla is implicated in various pathological conditions, particularly in a wide range of cancers (Luo et al., 2022; Yu et al., 2021; Miao et al., 2023; Jiang et al., 2021). For instance, aberrant lactylation has been shown to sustain tumorigenic pathways such as hypoxia-inducible factor 1-alpha (HIF-1α) signaling, enhance glycolytic metabolic reprogramming, and promote angiogenesis and immune evasion (Yu et al., 2021). Furthermore, Kla also occurs on non-histone proteins, particularly metabolic enzymes, where it facilitates metabolic reprogramming in tumor microenvironments (Yang et al., 2022; Chen et al., 2024).

Accumulating evidence indicates that Kla plays an important role in the development and progression of CVDs (Zhang et al., 2023; Ma et al., 2024; Wang et al., 2023a). Lactate, once regarded merely as a glycolytic byproduct, is now recognized as a major oxidative substrate and signaling molecule in the cardiovascular system, linking cellular metabolic status to gene regulation (Lopaschuk et al., 2021; Murashige et al., 2020; Brooks, 2020). Recent studies have further demonstrated that lactate can modulate inflammatory responses through Kla, including epigenetic regulation of immune activation and macrophage polarization (Zhang et al., 2019; Chen et al., 2021a). Given that chronic inflammation is a central driver of endothelial dysfunction and atherosclerosis, these findings suggest a mechanistic contribution of Kla to CVDs pathogenesis. Lactate has also been shown to promote vascular smooth muscle cell phenotypic switching and vascular remodeling (Yang et al., 2017); however, the specific role of Kla in endothelial cells remains largely undefined. Considering the well-established association between Pg infection, systemic inflammation, and cardiovascular complications, we hypothesize that Pg-induced lactylation reprogramming may represent a previously unrecognized mechanism linking periodontal infection to CVDs progression.

In this study, we employed quantitative lactylome profiling to comprehensively characterize Pg-induced lactylation changes in HUVECs. Our results reveal extensive lactylation reprogramming affecting multiple endothelial pathways and provide mechanistic insights into how periodontal infection may contribute to cardiovascular pathogenesis.

## 2 Materials and methods

### 2.1 Porphyromonas gingivalis culture

The *Porphyromonas gingivalis* W83 strain (sourced from the State Key Laboratory of Oral Diseases, West China Hospital of Stomatology, Sichuan University, China) was initially cultured on anaerobic blood agar plates containing 5% defibrinated sheep blood (Yikang Life Sciences, China).

Incubation was carried out at 37°C for 5–7 days until distinct black colonies became visible. Subsequently, the bacterial colonies were passaged twice: each passage involved overnight cultivation at 37°C under strict anaerobic conditions in brain heart infusion (BHI) broth (Becton, Dickinson and Company, USA), which was pre-supplemented with hemin (5 µg/mL) and vitamin K (5 µg/mL).

For the monitoring of bacterial growth, overnight cultures were prepared, and the optical density (OD) of the Pg suspension was determined at a wavelength of 600 nm (OD_600_). When the OD_600_ value reached 0.6 (corresponding to the mid-logarithmic growth phase), the number of colony-forming units (CFU) was enumerated via the standard plate count method, yielding a bacterial concentration of approximately 10^9^ CFU/mL.

### 2.2 Human umbilical vein endothelial cells culture and interaction of Pg with HUVECs

Human umbilical vein endothelial cells (provided by West China Hospital of Stomatology, State Key Laboratory of Oral Diseases, Sichuan University, China) were maintained in Dulbecco’s modified Eagle’s medium (DMEM; Gibco, USA) containing 10% fetal bovine serum (FBS) and 1% penicillin-streptomycin (Gibco, USA) under standard culture conditions (37°C, 5% CO_2_, humidified atmosphere).

Cells were seeded at a density of 10^6^ cells/mL following quantification using a Coulter Counter. For infection experiments, HUVECs were cultured in six-well flat-bottom plates (15.5 mL/well volume; 9.6 cm²/well surface area) until confluence. Pg strains were grown to mid-log phase (OD_600_ = 0.6), harvested by centrifugation, washed twice with PBS, and resuspended in antibiotic-free DMEM at a final concentration of 10^8^ CFU/mL. Bacteria were added to confluent HUVEC monolayers at a multiplicity of infection (MOI) of 100 and incubated for 2, 4, and 6 hours at 37°C with 5% CO_2_. Uninfected HUVECs served as the control group.

### 2.3 Protein extraction and western blot

HUVECs, co-cultured with or without Pg, were harvested by scraping and washed twice with ice-cold PBS. Total protein was extracted using cell lysis buffer (P0013, Beyotime Biotechnology, China) containing 1% protease inhibitor (K1007, Ape Bio, USA) and deacetylase inhibitor (K1017, Ape Bio, USA). Cell lysis was achieved by sonication using an ultrasonic probe. Protein concentrations in the extracts were subsequently quantified via the bicinchoninic acid (BCA) assay (P0010S, Beyotime Biotechnology, China) following the manufacturer’s protocols.

Equal amounts of protein samples were loaded and separated by sodium dodecyl sulfate-polyacrylamide gel electrophoresis (SDS-PAGE) using 12% gels, then transferred to nitrocellulose membranes (HATF00010, Merck Millipore, USA). Membranes were blocked for 1 hour in Tris-buffered saline with Tween-20 (TBST; 25 mM Tris-HCl, pH 8.0, 150 mM NaCl, 0.1% Tween-20) containing 5% non-fat dry milk, followed by overnight incubation at 4°C with a pan-Kla monoclonal antibody (PTM-1401, PTM BioLab, Hangzhou, China) diluted 1:1000 in blocking buffer. After three washes with TBST, the membranes were incubated with HRP-conjugated goat anti-mouse IgG secondary antibody (1:5000 in TBST; Thermo Fisher Scientific, USA) for 1 h at room temperature. Following six additional washes, protein bands were visualized using an enhanced chemiluminescence (ECL) substrate kit (Millipore, USA). Coomassie brilliant blue (CBB) was used as loading control.

### 2.4 Lactate quantification assay

Collected cells were washed twice with PBS and centrifuged at 1000 × g for 5 minutes to pellet the cells. The cell pellet was resuspended in 0.3–0.5 mL of isotonic PBS buffer (0.1 M, pH 7.4). Cells were then lysed by sonication (300 W or 20% amplitude, 5 s on/15 s off, repeated for 3–5 min) or manual homogenization. The homogenate was centrifuged at 4000 × g for 10 minutes, and the resulting supernatant was collected for lactate measurement according to manufacturer’s instruction (A019-2-1, Nanjing jiancheng, China).

### 2.5 Protein extraction and enzyme digestion

HUVEC samples were retrieved from the -80°C freezer and thawed on ice. A pre-prepared lysis buffer containing 8 M urea and 1% protease inhibitor was gradually added to the samples, followed by cell disruption via sonication using an ultrasonic homogenizer. The lysates were centrifuged at 15,000 × g for 10 minutes at 4°C to remove cellular debris. The supernatant was carefully collected into a new centrifuge tube, and its protein concentration was measured using the BCA assay.

Based on the quantified protein levels, equal amounts of protein from each sample were aliquoted, and the volume was normalized with additional lysis buffer. Trichloroacetic acid (TCA) was slowly introduced to a final concentration of 20%, and the mixture was vortexed thoroughly before incubation at 4°C for 2 hours to facilitate protein precipitation. After centrifugation (4°C, 15,000 × g, 5 minutes), the precipitated proteins were collected and subjected to three washes with pre-chilled acetone at -20°C. Following acetone evaporation, the pellet was resuspended in 100 mM tetraethylammonium bromide (TEAB) and dispersed via water bath sonication. Trypsin digestion was carried out overnight at 37°C using an enzyme-to-protein ratio of 1:50 (w/w).

Reduction was achieved by treatment with 5 mM dithiothreitol (DTT) at 56°C for 30 minutes, followed by alkylation with 15 mM iodoacetamide (IAA) for 15 minutes at room temperature in the dark. After the alkylation step, the pH of the samples was adjusted to 2–3 using 10% trifluoroacetic acid (TFA). Desalting was performed with a Strata X column (Phenomenex), and the samples were subsequently lyophilized. The dried peptides were reconstituted in water and quantified using the Pierce™ Quantitative Peptide Assays & Standards kit (Thermo Fisher Scientific, USA).

### 2.6 Affinity enrichment

The lysine-lactylated (Kla) peptides were enriched using anti-lactyl-lysine antibody-conjugated agarose beads (WM102, Micrometer Biotech, Hangzhou, China). Briefly, tryptic peptides were dissolved in NETN buffer (50 mM Tris−HCl pH 8.0, 100 mM NaCl, 1 mM EDTA, 0.5% NP−40) and incubated with the beads overnight at 4°C with gentle agitation. After incubation, the beads were washed three times with NETN buffer and once with deionized water to remove non−specific bindings. The bound peptides were then eluted with 0.1% trifluoroacetic acid (TFA), desalted using C18 ZipTips (Millipore), and dried under vacuum prior to LC−MS/MS analysis.

### 2.7 Peptides analysis by liquid chromatography-tandem mass spectrometry

Chromatographic separation was performed using a nanoflow Vanquish Neo system (Thermo Fisher Scientific). Subsequently, the eluted samples were analyzed via Data-Independent Acquisition (DIA) mass spectrometry on an Astral high-resolution mass spectrometer (Thermo Scientific), operated in positive ion mode. The precursor ion scan range was set from 380 to 980 m/z. Full MS scans were acquired at a resolution of 240,000 at 200 m/z, with a normalized AGC target of 500%- a parameter of Obitrap mass spectrometer, which controls the number of ions in the Orbitrap by filling the C-trap for a calculated period of time, and a maximum injection time of 5 ms. For DIA-MS^2^, 150 scan windows were defined with a 4 m/z isolation width. Higher-Energy Collisional Dissociation (HCD) fragmentation was carried out at 25 eV collision energy. The AGC target was set to 500% with a maximum ion injection time of 10 ms.

### 2.8 Database search

The DIA data was processed using the DIA-NN software. The software parameters were set as follows: the enzyme used was trypsin, with a maximum missed cleavage site set to 1. Fixed modification was Carbamidomethyl (C), and dynamic modifications were set to Lactylation (K). Proteins identified through database retrieval were required to pass the set filtering parameter of FDR < 1%. Fixed modification is necessary for cysteine (alkylation) after disulfide bond reduced with DTT and alkylated with IAA (99-100% percent), and dynamic modification is mainly searched against K for lactylation (72 mass shift), which is the common amino acid that lacytylation happens.

### 2.9 Protein annotation and functional enrichment

Protein annotation and functional enrichment were performed as previously described (Li et al., 2023b). For Gene Ontology (GO) annotation, GO terms were assigned to the identified proteins using eggnog-mapper against the EggNOG database (http://eggnog5.embl.de/#/app/home). The annotated proteins were subsequently categorized based on cellular components (CC), molecular functions (MF), and biological processes (BP). Domain annotation was performed using PfamScan via protein sequence alignment against the Pfam database (https://www.ebi.ac.uk/interpro/entry/pfam/#table). Pathway analysis was carried out with the Kyoto Encyclopedia of Genes and Genomes (KEGG) database (http://www.kegg.jp/kegg/mapper.html). Subcellular localization predictions were generated using the eukaryote-specific tool WolF Psort.

For each functional category, a two-tailed Fisher’s exact test was applied to evaluate the enrichment significance of differentially expressed proteins against the background of all identified proteins. Terms with an adjusted p-value of less than 0.05 were considered statistically significant.

### 2.10 Cluster analysis based on protein functional enrichment

To investigate conserved and divergent functional patterns-such as Gene Ontology (GO) terms, KEGG pathways, and protein domains-across multiple comparison groups, cluster analysis was applied to the functional enrichment results of differentially expressed proteins (DEPs) from each group. Significantly enriched terms were first selected using a threshold of p < 0.05 in at least one comparison group. The resulting matrix of p-values was subjected to a -log_10_ transformation, followed by Z-normalization within each functional category. Hierarchical clustering was then performed using Euclidean distance and average linkage. Finally, resulting clusters were visualized in a heatmap generated with the R package “pheatmap”.

### 2.11 Protein-protein interaction

To construct a protein-protein interaction (PPI) network, the accessions or sequences of differentially expressed proteins identified from comparative analyses were queried against the STRING database (version 12.0). Interactions with a confidence score greater than 0.7 were retained as high-confidence PPIs. The resulting network was visualized using the “visNetwork” R package. Highly interconnected clusters within the network were identified using the MCODE plugin, and the overall PPI network was visualized and further analyzed with Cytoscape (version 3.9.1).

### 2.12 Motif analysis

All identified Kla substrates in HUVECs were used to analyze the flanking sequences at sites of Kla with Motif-X algorithm-based MoMo software. Ten neighboring amino acid residues on each side of the modification site were selected as the positive set.

### 2.13 Statistical analysis

In the proteomic analysis, relative quantification of each site/protein across groups was analyzed using Student’s t-test to evaluate statistical significance. Enrichment of differentially expressed proteins relative to all identified proteins was assessed for all functional categories via Fisher’s exact test. A significance threshold of adjust p < 0.05 was applied to both tests. In lactate measurement, statistical significance was determined using one-way analysis of variance (ANOVA) followed by Tukey’s multiple comparisons test. In all experiments, there were 3 biological replicates per group.

## 3 Results

### 3.1 *Pg* infection induces time-dependent lysine lactylation changes in HUVECs

**Figure 1:**
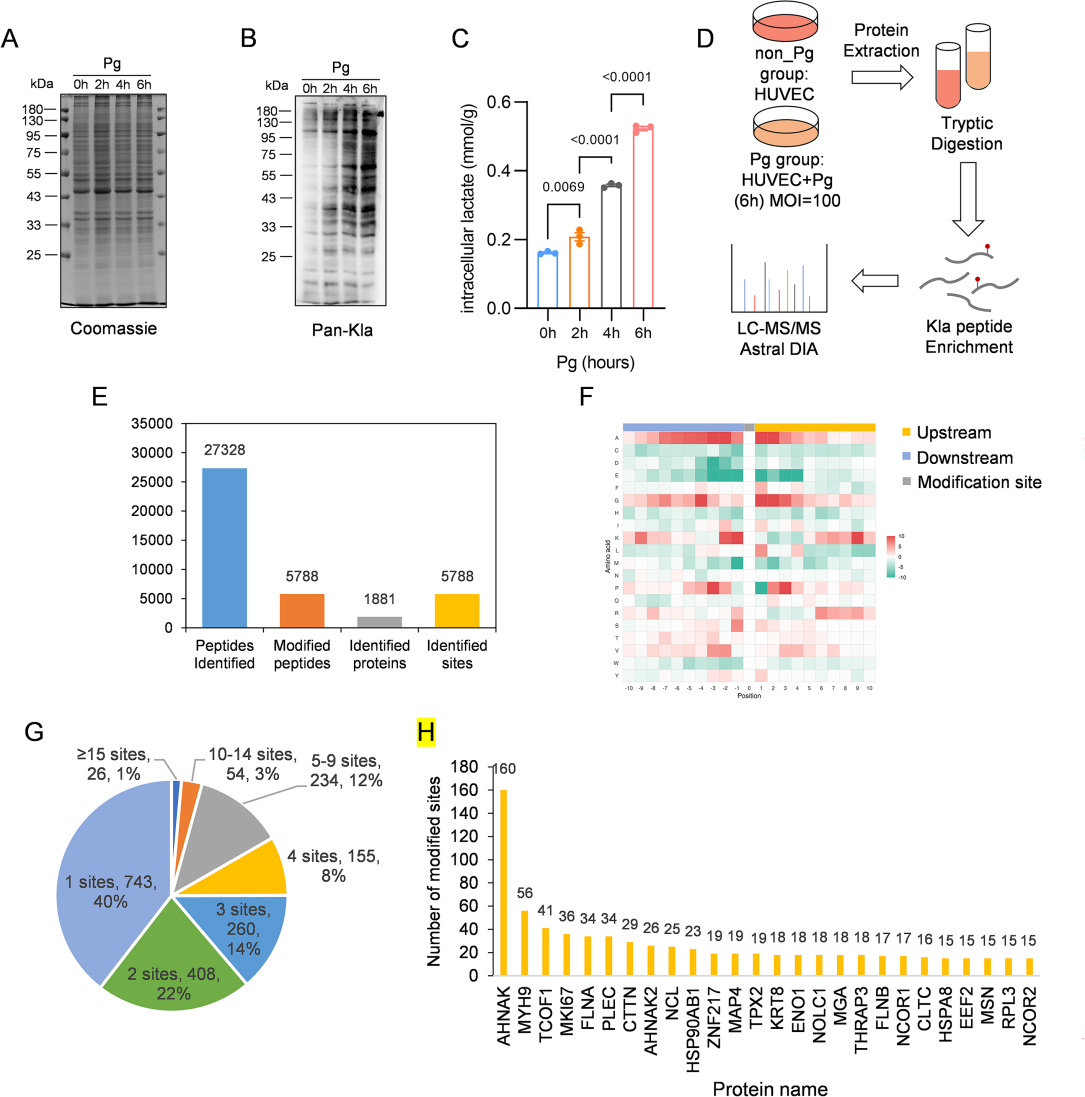
The lysine lactylome profiling in Pg-infected HUVECs. **(A)** Coomassie brilliant blue R-250 staining of SDS-PAGE gel as the loading control. **(B)** Immunoblot analysis of lactylated proteins using an antibody specific for lactylated lysine residues (pan-Kla) in lysates from HUVECs with or without Pg infection. **(C)** Quantification of intracellular lactate in HUVECs at different time points post-infection. Data are means ± SEM; n = 3 biological replicates; statistical significance was determined using one-way analysis of variance (ANOVA) followed by Tukey’s multiple comparisons test. **(D)** Multi-omics workflow. **(E)** General information of Kla proteins and sites. **(F)** Motif analysis of all identified Kla proteins. Red indicates high frequency and green means low frequency. **(G)** The number of lactylated sites identified per protein in HUVECs. **(H)** Frequency distribution of proteins with different numbers of Kla sites.

To investigate whether Pg infection alters protein lactylation in endothelial cells, we first examined global Kla levels at different time points post-infection. Western blot analysis using pan-Kla antibody showed a time-dependent increase in global Kla from 2 to 6 h post-infection (**Figures 1A, B**), accompanied by a parallel rise in intracellular lactate (**Figure 1C**). These coordinated changes indicate that Pg infection may trigger glycolytic activation and metabolite accumulation that may drive subsequent lactylation.

### 3.2 Lactylome profiling reveals extensive modifications in HUVECs

To systematically characterize the lactylome landscape, we performed quantitative proteomics analysis comparing Pg-infected (6h) and control HUVECs. Total proteins from HUVECs were extracted, and a proteomic research strategy involving affinity enrichment using anti-lactyl-lysine antibody-conjugated agarose beads and nano LC-MS/MS was employed for Kla-modified protein identification, with three biological replicates performed for verification (**Figure 1D**). After conducting three repeated tests, we identified 5788 Kla sites spanning 1881 proteins (**Figure 1E**) using a highly conservative threshold (FDR < 1%) (**Supplementary Table 1**). To characterize the substrate specificity of Kla sites, the amino acid sequences flanking each identified lactylation site (from −10 to +10) were analyzed against the entire HUVECs proteomic background using the Motif-X algorithm (Schwartz and Gygi, 2005). The positional frequency heatmap (**Figure 1F**) revealed a highly conserved preference for small, flexible amino acids flanking the central lysine. Specifically, alanine (A) and glycine (G) were significantly enriched across the positions from -10 to +10. Conversely, several amino acids, including the charged residues aspartate (D), glutamate (E), and histidine (H); the sulfur-containing cysteine (C) and methionine (M); anTCOFd the bulky hydrophobic tryptophan (W), were consistently depleted in the same regions. This pronounced sequence bias suggests that lactylation occurs preferentially in structurally accessible protein regions characterized by minimal steric hindrance. This modification pattern shares some similarities with prior reports on protein lactylation (Gao et al., 2020; Zhu et al., 2023).

Approximately 743 proteins (40%) contained only one Kla site, whereas 26 proteins (∼1%) were identified as highly modified, each exhibiting more than 15 sites (**Figure 1G**). Among the most heterogeneously modified proteins in HUVECs were neuroblast differentiation-associated protein (AHNAK), myosin-9 (MYH9), treacle protein (TCOF1), FHA domain-interacting nucleolar phosphoprotein (MKI67), filamin-A (FLNA), and plectin (PLEC). Each of these contained over 30 lactylation sites, with AHNAK being the most extensively modified (160 sites) (**Figure 1H**). Details of proteins harboring more than fifteen modification sites are presented in **Table 1**. These highly modified proteins are primarily involved in cytoskeletal organization, nuclear structure, and cellular adhesion processes critical for endothelial barrier function.

**Table 1:** The lactylated proteins with more than 15 modification sites.

Protein accession	Gene name	Protein description	Number of Kla sites	Protein function
Q09666	AHNAK	Neuroblast differentiation-associated protein AHNAK	160	A large scaffold protein involved in cytoskeletal organization, cell membrane stability, and signal transduction
P35579	MYH9	Myosin-9	56	A non-muscle myosin involved in actin cytoskeleton dynamics, cell contractility, and migration
Q13428	TCOF1	Treacle protein	41	A nucleolar protein required for ribosome biogenesis and regulation of RNA polymerase I transcription
P46013	MKI67	Proliferation marker protein Ki-67	36	A nuclear protein associated with cell proliferation and cell cycle progression
P21333	FLNA	Filamin-A	34	An actin-binding protein that crosslinks actin filaments and regulates cell shape and mechanotransduction
Q15149	PLEC	Plectin	34	A cytolinker protein that connects intermediate filaments to actin and microtubules, maintaining cellular integrity
Q14247	CTTN	Src substrate cortactin	29	An actin-binding protein that regulates cytoskeletal remodeling and cell motility
Q8IVF2	AHNAK2	protein AHNAK2	26	A large structural protein involved in cytoskeletal organization and cellular architecture
P19338	NCL	Nucleolin	25	A multifunctional nucleolar protein involved in ribosome biogenesis, RNA metabolism, and cell proliferation
P08238	HSP90AB1	Heat shock protein HSP 90-beta	23	A molecular chaperone that stabilizes and regulates the activity of multiple client proteins
O75362	ZNF217	Zinc finger protein 217	19	A transcriptional regulator involved in chromatin remodeling and gene expression control
P27816	MAP4	Microtubule-associated protein 4	19	A microtubule-associated protein that regulates microtubule stability and organizatio
Q9ULW0	TPX2	Targeting protein for Xklp2	19	A spindle assembly factor essential for microtubule nucleation and mitotic progression
P05787	KRT8	Keratin, type II cytoskeletal 8	18	An intermediate filament protein that provides structural support in epithelial cells
P06733	ENO1	Alpha-enolase	18	A glycolytic enzyme that catalyzes the conversion of 2-phosphoglycerate to phosphoenolpyruvate
Q14978	NOLC1	Nucleolar and coiled-body phosphoprotein 1	18	A nucleolar phosphoprotein involved in rRNA transcription and ribosome assembly
Q8IWI9	MGA	MAX gene-associated protein	18	A transcription factor that regulates gene expression through interaction with MAX
Q9Y2W1	THRAP3	Thyroid hormone receptor-associated protein 3	18	A nuclear protein involved in transcriptional regulation and RNA processing
O75369	FLNB	Filamin-B	17	An actin-binding protein that organizes the cytoskeleton and regulates cell migration
O75376	NCOR1	Nuclear receptor corepressor 1	17	A transcriptional corepressor that regulates gene expression by recruiting histone deacetylases
Q00610	CLTC	Clathrin heavy chain 1	16	A clathrin heavy chain protein involved in vesicle formation and intracellular trafficking
P11142	HSPA8	Heat shock cognate 71 kDa protein	15	A constitutively expressed heat shock protein involved in protein folding and quality control
P13639	EEF2	Elongation factor 2	15	A translation elongation factor essential for protein synthesis
P26038	MSN	Moesin	15	An ERM family protein that links the actin cytoskeleton to the plasma membrane
P39023	RPL3	Large ribosomal subunit protein uL3	15	A ribosomal protein that is a component of the large ribosomal subunit and involved in protein translation
Q9Y618	NCOR2	Nuclear receptor corepressor 2	15	A transcriptional corepressor involved in epigenetic regulation of gene expression

### 3.3 Pg infection reprograms the functional lactylation landscape

**Figure 2:**
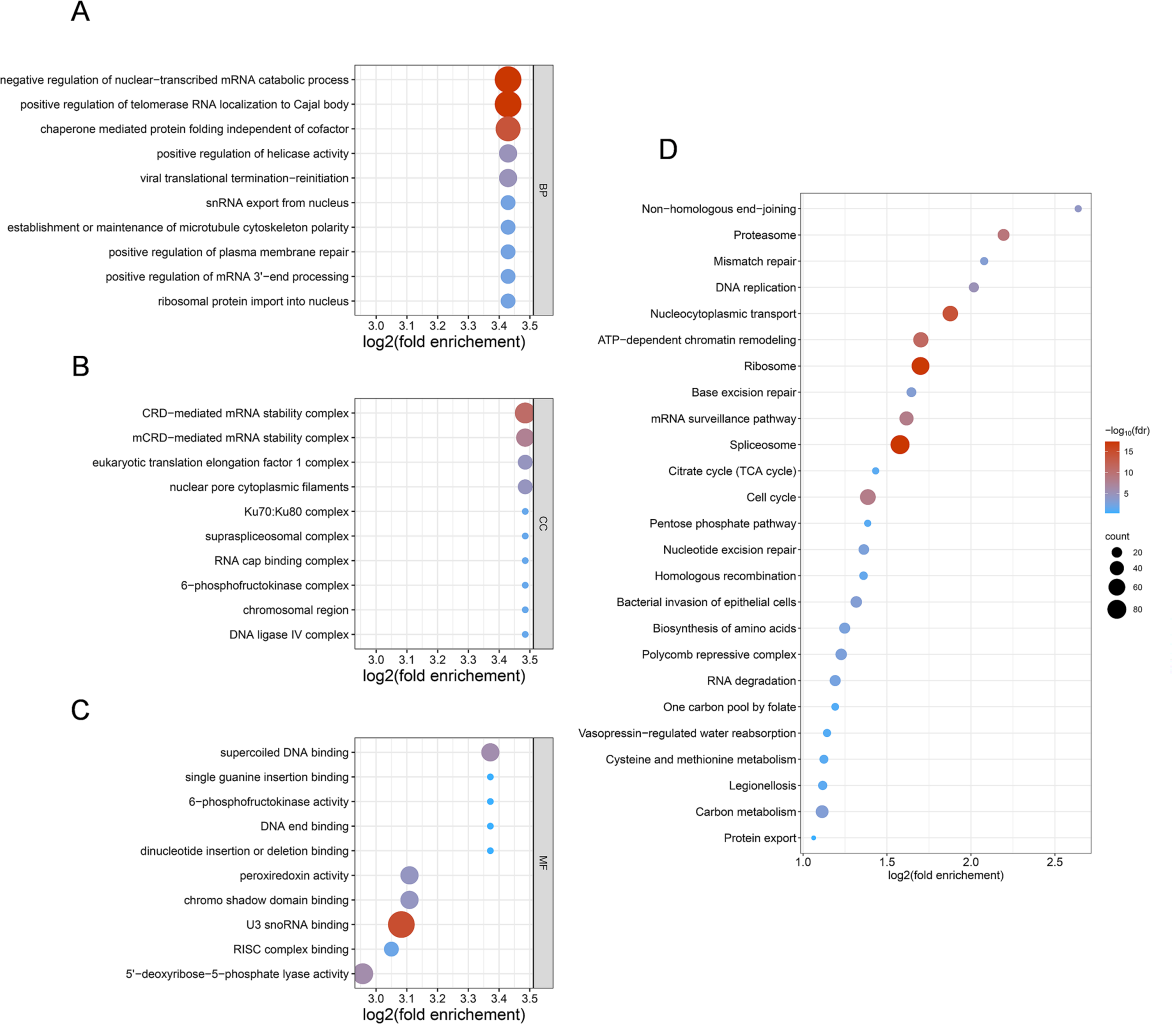
Pg infection reprograms the functional lactylation landscape. Top 10 enriched GO terms associated with lysine lactylation (Kla) sites: **(A)** Biological process (BP). **(B)** Cellular component (CC). **(C)** Molecular function (MF). **(D)** KEGG enrichment analysis of modified proteins. For each category, a two-tailed Fisher’s exact test was used to test the enrichment of the identified modified proteins against all proteins in the species database. Fold enrichment ≥ 1.5 and adjusted P values ≤ 0.05 were considered significant. Bubble color represents the statistical significance of the enrichment, expressed as -log10(FDR). A gradient from blue to red indicates increasing significance, with red bubbles denoting the most highly enriched pathways. All data are based on lactylation analysis performed on three biological replicates of HUVECs lysates with or without Pg infection.

To explore the role of Kla in regulating the cellular physiological processes of HUVECs, we performed functional enrichment analysis on the Kla-modified substrates identified in this study by using the Database for Annotation, Visualization, and Integrated Discovery (https://davidbioinformatics.nih.gov/, DAVID). The 1,881 identified lactylated protein sequences were uploaded to DAVID database. Results were ranked by fold enrichment, which reflects the degree to which lactylated proteins are concentrated in a given functional category compared to the whole proteome background, and the top 10 terms from the GO analysis and the top 25 pathways from the KEGG analysis were selected for visualization, as shown in **Figure 2**. GO enrichment analysis revealed significant enrichment of lactylated proteins in key biological processes, including mRNA catabolic regulation, translational control, DNA repair, and microtubule polarity maintenance (**Figure 2A**). Cellular Component analysis indicated strong localization of these proteins to multifunctional complexes involved in gene expression, DNA repair, and metabolic regulation—such as nuclear pore cytoplasmic filaments, the supraspliceosomal complex, and the Ku protein complex (Ku70/Ku80) (**Figure 2B**). Furthermore, enrichment of translation elongation and glycolytic complexes (e.g., the eukaryotic translation elongation factor 1 complex and 6-phosphofructokinase complex) suggests that lactylation may function as a regulatory modification linking cellular metabolism to transcriptional and translational processes in HUVECs. Molecular Function analysis also identified significant enrichment in terms including nucleic acid binding, kinase activity, and peroxiredoxin activity (**Figure 2C**).

According to KEGG pathway enrichment (**Figure 2D**), the top enriched five pathways were non-homologous end joining (FC = 6.23, P = 5.33^10^-5^), proteasome (FC = 4.58, P = 3.58^10^-10^), mismatch repair (FC = 4.22, P = 3.85^10^-4^), DNA replication (FC = 4.05, P = 6.82^10^-6^), nucleocytoplasmic transport (FC = 3.67, P = 5.53^10^-15^). These findings suggest that lactylation may serve as a regulator to modulate DNA damage response, protein turnover, and gene expression in HUVECs, thereby providing potential insights into the cellular response mechanisms of HUVECs following bacterial infection. Details of functional enrichment analysis are listed in **Supplementary Table 2**.

### 3.4 Functional enrichment analysis reveals coordinated pathway alterations

**Figure 3:**
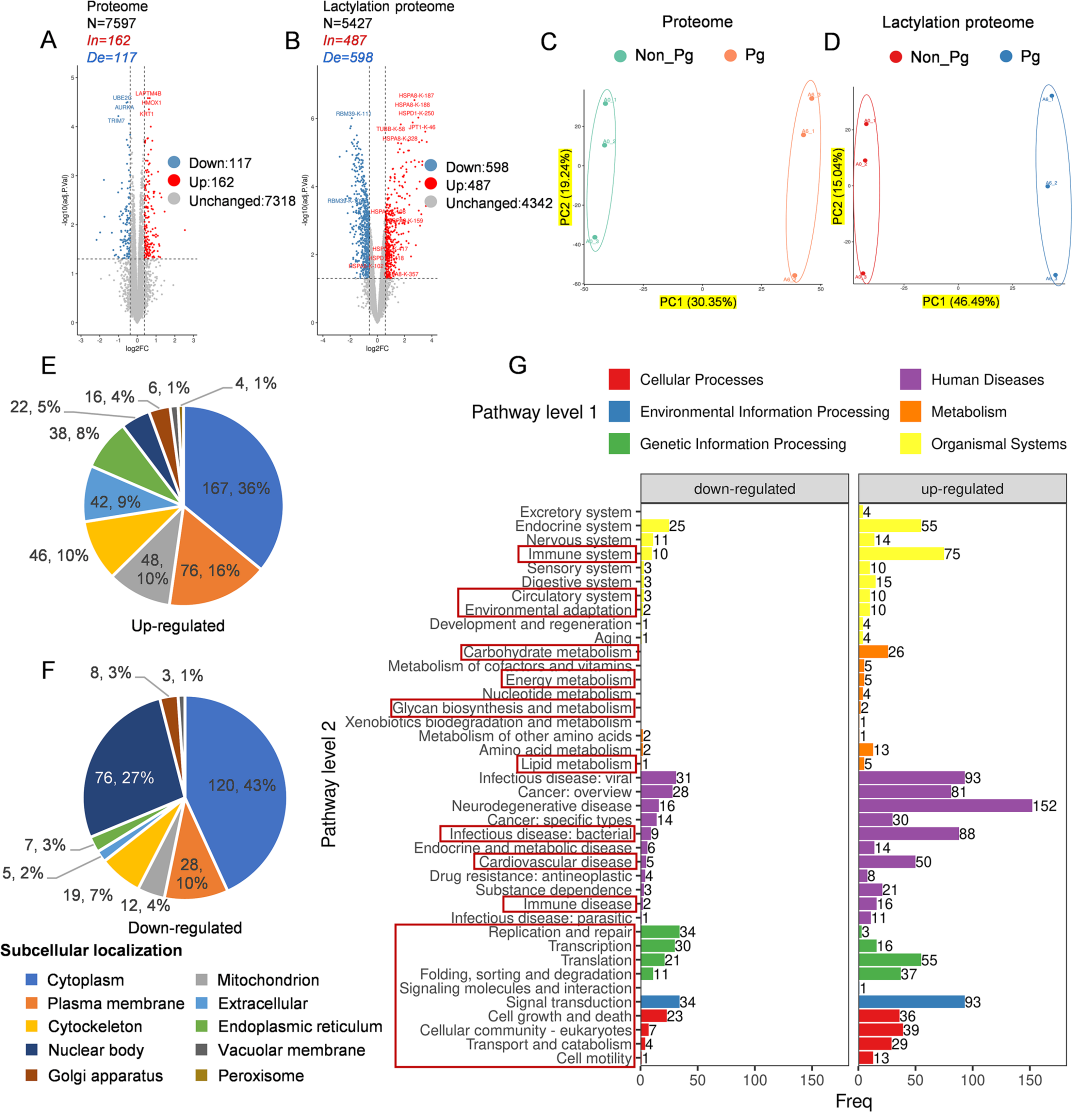
Functional enrichment analysis reveals coordinated pathway alterations. Volcano plots showing the differentially expressed **(A)** proteins and **(B)** lactylated proteins in Pg-infected compared with control group. Blue dots represent downregulated proteins, while red dots represent upregulated proteins. Principal component analysis of **(C)** proteome and **(D)** lactylation proteome. Subcellular localization prediction of modified proteins **(E)** up-regulated modified sites. **(F)** down-regulated modified sites. **(G)** KEGG-based classification of the differentially modified proteins. The red boxes highlight the key KEGG pathway groups most relevant to our study’s focus. For each category, a two-tailed Fisher’s exact test was used to test the enrichment of the identified modified proteins against all proteins in the species database. Fold enrichment ≥ 1.5 and adjusted P values ≤ 0.05 were considered significant. All data are based on lactylation analysis performed on three biological replicates of HUVECs lysates with each condition.

Quantitative proteomic analysis of HUVECs revealed extensive remodeling of the Kla landscape following infection with Pg (**Figure 3**). Among 7,597 proteins detected, 279 differentially expressed proteins (DEPs) were identified, including 162 upregulated and 117 downregulated proteins in response to infection (ratio (Pg/non_Pg) ≥ 1.5 or ≤ 0.67, respectively, and p ≤ 0.05) (**Figure 3A**, **Supplementary Table 3**). Concurrently, lactylomic profiling identified 1,085 significantly altered lactylation sites, with 487 sites increased and 598 sites decreased under the same significance thresholds (**Figure 3B**, **Supplementary Table 4**). Notably, some proteins exhibited extensive site-specific modulation; for example, the structural protein AHNAK contained 37 differentially modified lysines, of which 22 were up-regulated and 15 were down-regulated. Principal component analysis (PCA) demonstrated clear separation between infected and control groups in both the proteome (**Figure 3C**) and lactylome (**Figure 3D**), indicating substantial changes in both protein abundance and lactylation modification status after Pg infection.

Subcellular localization analysis of Kla proteins in HUVECs revealed a broad yet distinct distribution pattern across multiple intracellular structures (**Figures 3E, F**, **Supplementary Table 5**). The majority of Kla-modified proteins were localized to the cytoplasm (287 proteins, 38.6%), followed by the cell membrane (104 proteins, 14.0%) and the nucleus (98 proteins, 13.2%). The cytoplasm is the primary site of lactate production via glycolysis (Li et al., 2023a), which directly supports the hypothesis that lactate generation serves as a direct sign of metabolic status (Li et al., 2025a). Kla modifications in the cytoplasm may enable rapid regulation of metabolic enzyme activity and signal transduction processes. Notably, the substantial presence of Kla targets in the nucleus further reinforces its emerging role as an epigenetic modification, which is capable of modulating gene expression programs in response to bacterial infection. Additionally, significant Kla levels were detected in mitochondria, the Golgi apparatus, the cytoskeleton, and the endoplasmic reticulum. This widespread distribution suggests that lysine lactylation may exert multifaceted roles in diverse intracellular processes, including metabolic regulation, structural organization, and nuclear functions.

To investigate the functional roles of these differentially modified proteins during Pg infection, we performed functional analyses of these proteins corresponding to the differentially modified sites. KEGG pathway-based classification of the lactylated proteins revealed their significant involvement in a wide spectrum of biological processes and disease-related pathways (**Figure 3G**, **Supplementary Table 6**). Major enriched categories included immune system, circulatory system, environmental adaptation, infectious diseases (bacterial), and cardiovascular diseases. Furthermore, lactylated proteins were also highly associated with core cellular functional clusters such as DNA replication and repair, gene transcription and translation, protein folding, sorting and degradation, as well as signal transduction.

### 3.5 Pg infection remodels the functional landscape of the lactylome in HUVECs

**Figure 4:**
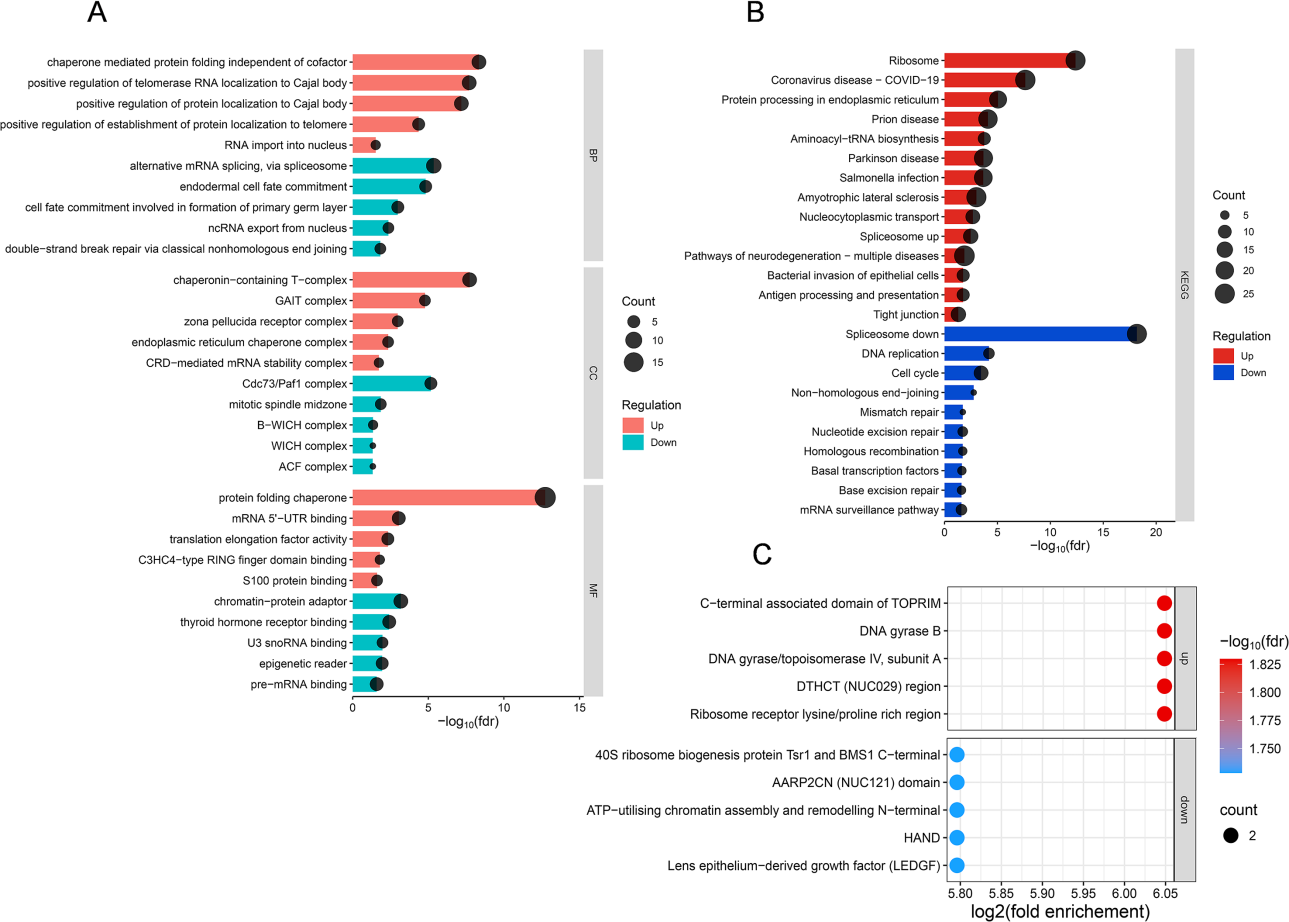
Pg infection remodels the functional landscape of the lactylome in HUVECs. **(A)** Bar chart of proteins with differentially Kla sites including BP, CC, MF based on GO enrichment analysis. The pink columns represent up-regulated sites, and the green column represent down-regulated sites. **(B)** Bar chart of proteins with differentially Kla sites based on KEGG enrichment analysis. The red columns represent up-regulated sites, and the blue column represent down-regulated sites. **(C)** Bubble chart of domain analysis through Pfam-based enrichment analysis presents in the modified proteins. For each category, a two-tailed Fisher’s exact test was used to test the enrichment of the identified modified proteins against all proteins in the species database. Fold enrichment ≥ 1.5 and adjusted P values ≤ 0.05 were considered significant. All data are based on lactylation analysis performed on three biological replicates of HUVECs lysates with each condition.

Functional analysis of differentially lactylated proteins revealed their broad involvement in diverse cellular processes and compartments, highlighting the extensive impact of Pg infection on the lactylation profile of HUVECs. Lactylated proteins were significantly associated with biological processes including protein folding, protein localization, RNA transport, and RNA localization. In terms of molecular function, these modified proteins were primarily implicated in chaperone-assisted protein folding and binding activities directed toward mRNA and S100 proteins (a family of calcium-binding proteins that act as damage-associated molecular patterns to amplify inflammation) (Leśniak and Filipek, 2024), among other cellular components (**Figure 4A**, **Supplementary Table 7**). Furthermore, cellular component enrichment showed that up-regulated lactylated proteins were enriched in complexes such as the gamma interferon activated inhibitor of translation (GAIT) complex, chaperonin-containing T-complex, annulate lamellae, C-terminal RNA-binding domain (CRD)-mediated mRNA stability complex, and zona pellucida receptor complex. In contrast, down-regulated lactylated proteins were predominantly localized to the ATP-utilizing chromatin assembly and remodeling factor (ACF) complex, WSTF-ISWI chromatin remodeling (WICH) complex, Cdc73/Paf1 complex, basal transcription factor WICH (B-WICH) complex, and the mitotic spindle midzone (**Figure 4A**, **Supplementary Table 7**). This marked spatial segregation suggests that lactylation may exert distinct and opposing regulatory effects on specialized functional complexes in response to bacterial infection.

KEGG pathway enrichment analysis of differentially lactylated proteins revealed clear functional differences between up- and down-regulated modified proteins (**Figure 4B**, **Supplementary Table 7**). Up-regulated lactylated proteins were significantly enriched in pathways related to aminoacyl-tRNA biosynthesis, ribosome, antigen processing and presentation, bacterial invasion of cells, nucleocytoplasmic transport, protein processing in endoplasmic reticulum, spliceosome and tight junction. In contrast, down-regulated lactylated proteins were predominantly associated with non-homologous end-joining, DNA replication, spliceosome, mismatch repair, homologous recombination, basal transcription factors, base excision repair, nucleotide excision repair, cell cycle, and mRNA surveillance pathway. These findings suggest that bacterial infection of HUVECs makes lactylation redirect the cells—from keeping basic homeostasis to coping with Pg infection.

Based on domain enrichment analysis performed using the Pfam database (**Figure 4C**, **Supplementary Table 7**), lysine lactylation (Kla) substrates were found to be significantly enriched in functional domains implicated in DNA metabolism. Notably, strong enrichment was observed in nuclease-related domains, including the TOPRIM domain (present in topoisomerases and primases) and DNA gyrase domains, which are essential for DNA cleavage, supercoiling maintenance, and structural dynamics. The prominence of these domains among lactylated proteins suggests that lactylation may serve as a novel regulatory mechanism influencing DNA topology and genomic integrity. This modification could potentially affect critical processes such as DNA replication, repair, and transcriptional regulation, thereby enabling cells to adapt to metabolic fluctuations or infectious challenges (Singh et al., 2020; Chan et al., 2015).

**Figure 5:**
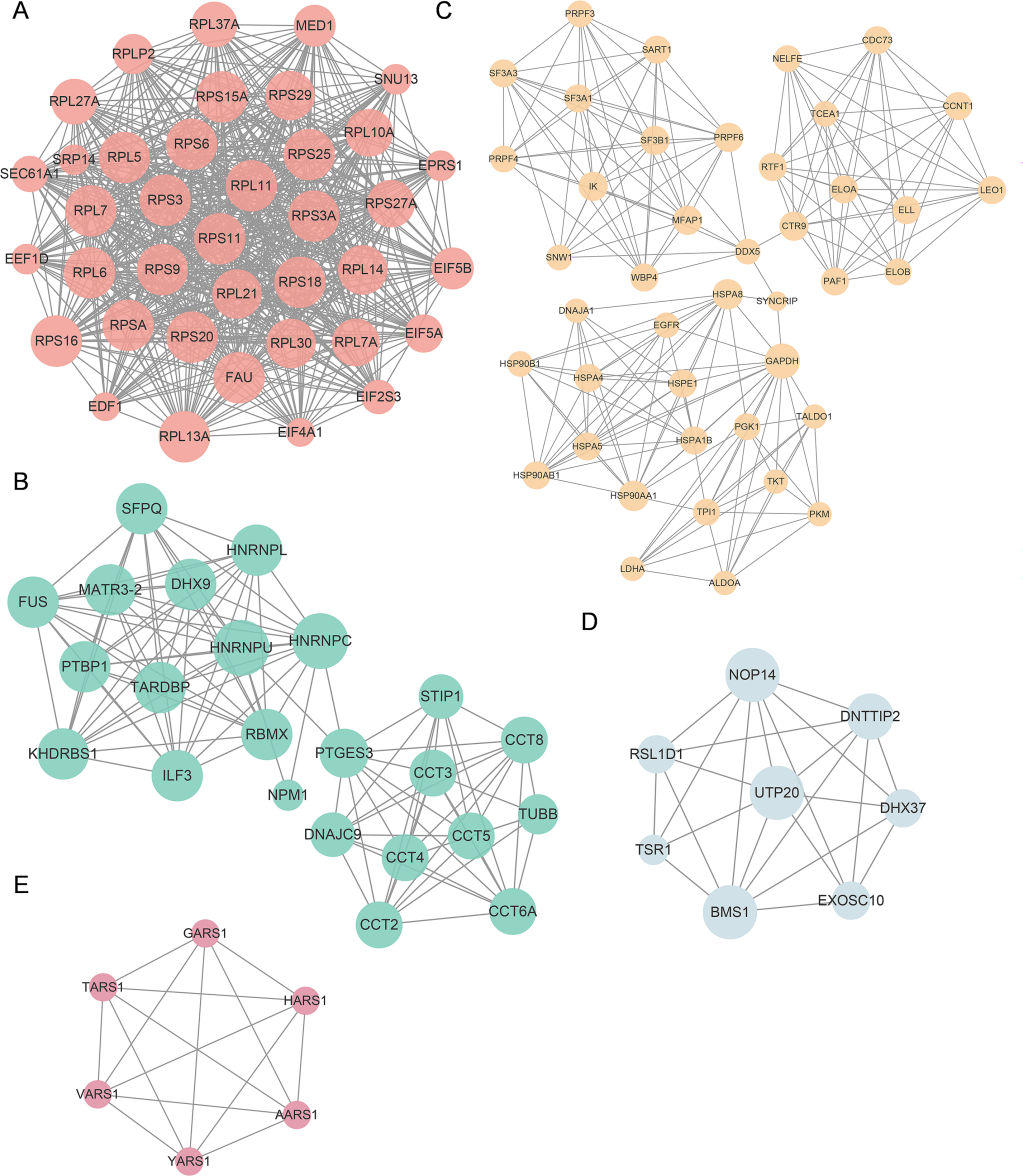
The top five clusters of highly interconnected lactylated PPI networks. **(A)** Cluster 1, enriched in ribosome pathway. MCODE score = 34.649, nodes = 38, edges = 641. **(B)** Cluster 2, enriched in spliceosome and proteins involved in mRNA surveillance pathways. MCODE score = 10.182, nodes = 23, edges = 112. **(C)** Cluster 3, enriched in spliceosome, metabolic processes, lipid metabolism and atherosclerosis-related pathways. MCODE score = 9.610, nodes = 42, edges = 197. **(D)** Cluster 4, enriched in rRNA processing. MCODE score = 6.571, nodes = 8, edges = 23. **(E)** Cluster 5, enriched in aminoacyl-tRNA biosynthesis. MCODE score = 6, nodes = 6, edges = 15. Nodes represent modified proteins. Node size and gene symbol size reflects interaction degree, RPS27A emerged as the hub molecule with 67 interactions.

Reversible post-translational modifications (PTMs) play an important role in dynamically remodeling protein–protein interaction (PPI) networks, thereby regulating essential cellular functions (Wang et al., 2022). In order to investigate the potential impact of lactylation on PPIs, we utilized the STRING database (Search Tool for the Retrieval of Interacting Genes/Proteins) (Szklarczyk et al., 2021) to reconstruct a global interaction network among the identified lactylated proteins (**Figure 5**). The resulting network was further analyzed using the molecular complex detection (MCODE) algorithm in Cytoscape (v3.10.1) to identify highly interconnected modules. This approach revealed eight significant high-density clusters (MCODE score≥4.5) (**Supplementary Table 8**), suggesting that lactylation may promote the formation of functionally cohesive protein complexes involved in specific biological processes.

Among the top five highly interconnected clusters identified through PPI network analysis, cluster 1 was predominantly composed of ribosome-associated proteins (**Figure 5A**, **Supplementary Figure 1A**), suggesting that lactylation may influence translational regulation and protein synthesis efficiency. Cluster 2 showed significant enrichment in spliceosome and proteins involved in mRNA surveillance pathways (**Figure 5B**, **Supplementary Figure 1B**), uncovering a potential role in pre-mRNA processing and RNA quality control. These findings are consistent with the KEGG pathway enrichment results, which also highlighted significant involvement of lactylated proteins in ribosome and spliceosome-related pathways (**Figure 4B**). Cluster 3 was primarily associated with the spliceosome, central metabolic processes—such as carbon metabolism and glycolysis/gluconeogenesis—as well as lipid metabolism and atherosclerosis-related pathways (**Figure 5C**, **Supplementary Figure 1C**), implying a link between lactylation and metabolic reprogramming in vascular endothelial cells. Cluster 4 was notably enriched in proteins participating in rRNA processing (**Figure 5D**, **Supplementary Figure 1D**), indicating a possible role of lactylation in ribosome biogenesis. Meanwhile, cluster 5 was significantly enriched in proteins associated with aminoacyl-tRNA biosynthesis (**Figure 5E**, **Supplementary Figure 1E**). Collectively, these cluster-specific profiles suggest that lysine lactylation significantly reorganizes protein interaction networks in HUVECs, thereby exerting profound effects on vital cellular processes including gene expression, metabolic homeostasis, and RNA processing.

## 4 Discussion

Studies have reported the detection of oral pathogenic bacteria, including Pg and *Aggregatibacter actinomycetemcomitans* (Aa) in lesion specimens from patients with CVDs such as atherosclerotic plaques (Brun et al., 2020; Mulhall et al., 2020) and infected heart valves in endocarditis (Radwan-Oczko et al., 2014). Notably, the abundance of Pg was significantly higher compared to other periodontal bacteria in these tissues (Joshi et al., 2021). Endothelial cells form the innermost layer of blood vessels and regulate critical physiological processes, including vascular contraction and dilation, inflammatory response mediation, coagulation and anticoagulation, and the control of vascular permeability (Trimm and Red-Horse, 2023). The onset and progression of CVDs, such as atherosclerosis and hypertension, are closely associated with endothelial dysfunction (Thayaparan et al., 2025). Therefore, in this study, we directly challenged HUVECs with Pg and examined alterations in Kla levels following infection. This experimental model summarizes key aspects of the pathophysiological processes underlying CVDs.

A major conceptual advance of this study is that lactylation changes induced by Pg follow the same time course as metabolic adaptation, consistent with a model in which lactylation acts as a dynamic and functionally impactful post-translational modification during host-pathogen interaction (Tyl et al., 2025; Li et al., 2024; Gu et al., 2024; Sun et al., 2024). The progressive increase in Kla signal over time suggests that lactylation represents a delayed host response, potentially linked to the accumulation of lactate (Li et al., 2023b). Bacterial infection is known to impose high energetic and biosynthetic demands on host cells (Yu et al., 2022; Zhao et al., 2025), often forcing a shift toward glycolysis even in oxygen-rich environments—a metabolic phenomenon similar to the Warburg effect observed in immune activation and tumor progression (Fendt, 2024; Zhang et al., 2019). Our findings indicate that endothelial cells adopt a comparable metabolic strategy under Pg infection. Here, increased lactylation represents an active signaling mechanism, where lactate is purposefully utilized to modify lysine residues, thereby changing the function of proteins involved in immunity, gene regulation, and vascular integrity, as reflected by the lactylation in structural and nuclear proteins such as AHNAK, FLNA, MYH9, and PLEC (Lee et al., 2023; Wang et al., 2023b; Ban and Shah, 2017; Koskeridis et al., 2024). This suggests that lactylation may serve as a form of metabolic memory, encoding sustained changes into lasting cellular responses.

The broad reconfiguration of the lactylome after Pg infection suggests a complex balance between host defense and cellular maintenance. Although not classical immune cells, endothelial cells function as semi-professional antigen-presenting cells and orchestrate leukocyte recruitment by releasing cytokines and expressing adhesion molecules (Pober et al., 2017). The enhanced lactylation of proteins linked to antigen processing and trafficking raises the possibility that lactylation contributes to endothelial–immune cross-talk, potentially amplifying inflammatory responses during periodontal bacteremia.

Structural and subcellular distribution of lactylation is another important implication of our findings. The strong modification of cytoskeletal and junctional proteins raises the possibility that lactylation may influence endothelial barrier permeability. Pg is known to disrupt endothelial junctions and promote trans-endothelial migration of inflammatory cells, thereby exacerbating systemic inflammation (Xie et al., 2020; Mekata et al., 2025). Given the central role of barrier function in vascular homeostasis, lactylation-driven modulation of cytoskeletal dynamics may contribute to the increased vascular permeability observed in patients with chronic periodontitis (Xiao et al., 2025). Furthermore, nuclear lactylation may coordinate transcriptional reprogramming through modifications on chromatin-associated proteins, potentially altering gene expression profiles involved in inflammation, angiogenesis, and coagulation.

To functionally classify the modified proteins, we reconstructed protein-protein interaction (PPI) networks using the STRING database and analyzed them with Cytoscape’s MCODE tool. The network analysis revealed that lactylation targets entire functional modules-particularly those involved in translation, RNA maturation, and metabolic flux-rather than individual proteins (**Figure 5**). This indicates that lactylation acts as a systems-level regulator, enabling endothelial cells to coordinate multiple processes rapidly in response to Pg infection. This coordinated remodeling aligns with recent findings in macrophages and tumor cells (Chen et al., 2022; Wan et al., 2024), where lactylation integrates energy metabolism with cellular responses. Our study extends this concept to vascular endothelial cells, suggesting that lactylation may be a common mechanism through which various stressors, such as infection, hypoxia, and metabolic overload, converge to alter shared signaling pathways.

The potential clinical implications of these findings are substantial. Periodontal disease has long been epidemiologically associated with cardiovascular diseases, yet the molecular pathways that connect oral bacteria to vascular pathology remain poorly understood. Our study identifies lactylation as a candidate pathway that could mediate this connection. For example, enhanced lactylation in antigen presentation pathways may amplify vascular inflammation, contributing to plaque formation and destabilization (Li et al., 2025b). These molecular signatures could form the basis for new biomarker panels to assess vascular risk in individuals with periodontal disease.

Despite these advances, several limitations warrant consideration. First, the study relies primarily on an *in vitro* endothelial model allowing for precise control of infection parameters, which cannot fully recapitulate the complex environment of human vasculature. Future validation in animal models of periodontitis-associated vascular inflammation will be essential to strengthen the physiological relevance of our findings. Second, while this study establishes the first comprehensive atlas of the endothelial lactylome under Pg infection, the specific functional consequences of individual lactylation events—such as the extensive modifications identified on AHNAK and MYH9—remain to be experimentally dissected. Subsequent studies employing site-directed mutagenesis are required to dissect whether lactylation activates or represses specific protein functions depending on the biochemical context. Third, it remains to be clarified whether the lactate driving these modifications is derived directly from Pg metabolic byproducts or solely through host metabolic reprogramming. Future investigations incorporating metabolic flux analysis or bacterial metabolic mutants will be necessary to resolve this host-pathogen metabolic crosstalk.

In conclusion, this study reveals that *Porphyromonas gingivalis* infection induces a comprehensive reorganization of endothelial lactylation, reshaping metabolic, epigenetic, and structural pathways that are fundamental to vascular health. These findings provide a conceptual framework for understanding how periodontal infections can exert systemic vascular effects and open new insights for developing lactylation-based diagnostic and therapeutic strategies.

## Data Availability

The dataset is publicly available under the accession identifier PXD073311. Direct link: https://proteomecentral.proteomexchange.org.
