# Supplementary material for: Porphyromonas gingivalis infection induces lysine lactylation reprogramming in human umbilical vein endothelial cells
Source: Front Cell Infect Microbiol. 2026 Jan 30;16:1706727. doi: 10.3389/fcimb.2026.1706727 (PMC12901344; doi:10.3389/fcimb.2026.1706727)
Supplement: Supplementary file 1 [file Table1.docx]

Supplementary Material

# Supplementary Tables

**Supplementary Table 1**. MS identified modification site detail

**Supplementary Table 2**. GO and KEGG enrichment of Kla substrates by DAVID database

**Supplementary Table 3**. DEPs list of proteome pg-vs-non_pg

**Supplementary Table 4**. DEPs list of lactylome pg-vs-non_pg

**Supplementary Table 5**. Subcell classify of pg-vs-non_pg

**Supplementary Table 6**. KEGG classify of pg-vs-non_pg

**Supplementary Table 7**. Functional enrichment of pg-vs-non_pg

**Supplementary Table 8.** PPI table

# Supplementary Figures


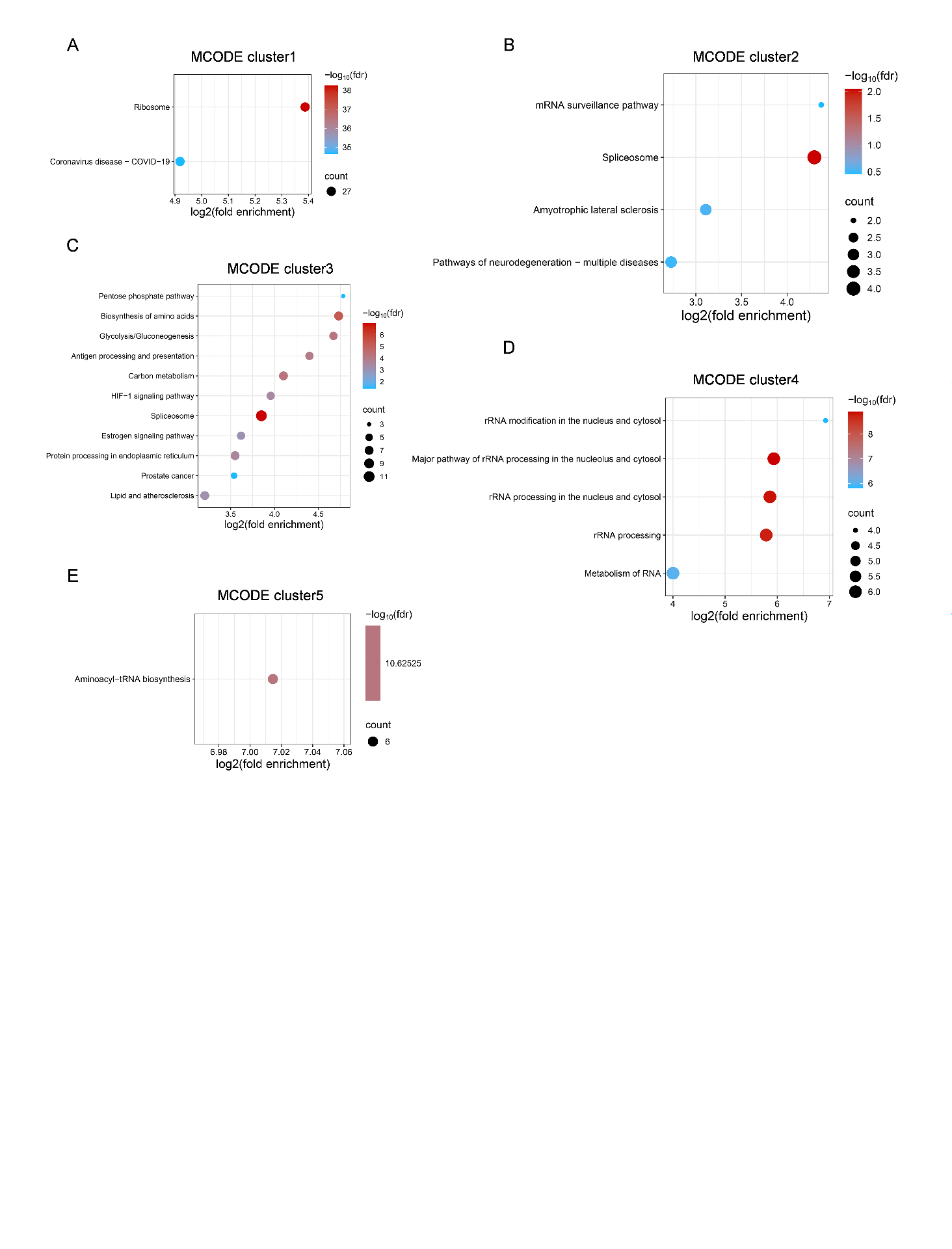


**Supplementary Figure 1**. KEGG enrichment analysis of the top five clusters of highly interconnected lactylated PPI networks. (A) Cluster 1, enriched in ribosome pathway. MCODE score = 34.649, nodes = 38, edges = 641. (B) Cluster 2, enriched in spliceosome and proteins involved in mRNA surveillance pathways. MCODE score = 10.182, nodes = 23, edges = 112. (C) Cluster 3, enriched in spliceosome, metabolic processes, lipid metabolism and atherosclerosis-related pathways. MCODE score = 9.610, nodes = 42, edges = 197. (D) Cluster 4, enriched in rRNA processing. MCODE score = 6.571, nodes = 8, edges = 23. (E) Cluster 5, enriched in aminoacyl-tRNA biosynthesis. MCODE score = 6, nodes = 6, edges = 15. Nodes represent modified proteins. Node size reflects the number of modified sites.
